# Supplementary material for: Fabrication of glipizide loaded polymeric microparticles; in-vitro and in-vivo evaluation
Source: PLoS One. 2025 Jan 9;20(1):e0313523. doi: 10.1371/journal.pone.0313523 (PMC11717267; doi:10.1371/journal.pone.0313523)
Supplement: Attachment — Submitted filename: Rebuttal Letter.docx [file pone.0313523.s001.docx]

Rebuttal Letter

| 1. Please ensure that your manuscript meets PLOS ONE's style requirements, including those for file naming. The PLOS ONE style templates can be found at | Thank you for the reminder. I have ensured that the manuscript complies with PLOS ONE's style requirements. The formatting of the main body, title, authors, and affiliations has been done according to the provided templates:   - [PLOS One Main Body Formatting Template](https://journals.plos.org/plosone/s/file?id=wjVg/PLOSOne_formatting_sample_main_body.pdf) - [PLOS One Title and Author Formatting Template](https://journals.plos.org/plosone/s/file?id=ba62/PLOSOne_formatting_sample_title_authors_affiliations.pdf)   Additionally, all file names are in accordance with PLOS ONE’s naming conventions.  If any further adjustments are required, please let me know. |
| --- | --- |
| 2. To comply with PLOS ONE submissions requirements, in your Methods section, please provide additional information regarding the experiments involving animals and ensure you have included details on (1) methods of sacrifice, (2) methods of anesthesia and/or analgesia, and (3) efforts to alleviate suffering. | **Methods of Sacrifice of Animals**: In accordance with standard ethical guidelines, no intentional sacrifice of animals was part of the study protocol. The study adhered to internationally accepted ethical principles for the care and use of animals in research. Any animal death that occurred during the study was an unintended and unfortunate event, likely related to the physiological effects of the substances administered, such as Alloxan monohydrate, which is known to induce significant physiological stress. At all times, the health and well-being of the animals were closely monitored by qualified personnel, and all efforts were made to prevent unnecessary harm. Additionally, the study was approved by the institutional Ethical Review Board (approval no. 205/QEC/GU), ensuring compliance with the necessary ethical standards.  **Methods of Anesthesia and/or Analgesia**: The study did not involve surgical or invasive procedures that would typically necessitate anesthesia. However, the administration of substances was conducted with utmost care to minimize any potential discomfort. Additionally, following ethical guidelines, if any animal had shown signs of distress beyond what was expected during the course of treatment, appropriate interventions would have been taken to mitigate discomfort, including analgesia or other supportive care measures as needed.  **Efforts to Alleviate Suffering**: Throughout the study, efforts were made to alleviate any potential suffering. For example, after the administration of Alloxan monohydrate to induce diabetes, animals were monitored for hypoglycemia, and oral glucose was administered to prevent severe hypoglycemic episodes. Moreover, the animals were under continuous supervision to ensure that any signs of undue distress were promptly addressed. The research team took all reasonable measures to safeguard the animals' well-being, consistent with ethical research standards. |
| 3. We note that your Data Availability Statement is currently as follows: [Data available in manuscript, if needed further feel free to contact]  Please confirm at this time whether or not your submission contains all raw data required to replicate the results of your study. Authors must share the “minimal data set” for their submission. PLOS defines the minimal data set to consist of the data required to replicate all study findings reported in the article, as well as related metadata and methods (https://journals.plos.org/plosone/s/data-availability#loc-minimal-data-set-definition).  For example, authors should submit the following data:  - The values behind the means, standard deviations and other measures reported;  - The values used to build graphs;  - The points extracted from images for analysis.  Authors do not need to submit their entire data set if only a portion of the data was used in the reported study.  If your submission does not contain these data, please either upload them as Supporting Information files or deposit them to a stable, public repository and provide us with the relevant URLs, DOIs, or accession numbers. For a list of recommended repositories, please see https://journals.plos.org/plosone/s/recommended-repositories.  If there are ethical or legal restrictions on sharing a de-identified data set, please explain them in detail (e.g., data contain potentially sensitive information, data are owned by a third-party organization, etc.) and who has imposed them (e.g., an ethics committee). Please also provide contact information for a data access committee, ethics committee, or other institutional body to which data requests may be sent. If data are owned by a third party, please indicate how others may request data access. | Yes, the submission contains all raw data necessary to replicate the findings of the study. This includes the values behind the means, standard deviations, and other statistical measures, as well as the data used to construct graphs. All data is included within the manuscript. Should additional data be required, we will make it available as Supporting Information files. There are no ethical or legal restrictions preventing the sharing of the data. |
| 4. PLOS requires an ORCID iD for the corresponding author in Editorial Manager on papers submitted after December 6th, 2016. Please ensure that you have an ORCID iD and that it is validated in Editorial Manager. To do this, go to ‘Update my Information’ (in the upper left-hand corner of the main menu), and click on the Fetch/Validate link next to the ORCID field. This will take you to the ORCID site and allow you to create a new iD or authenticate a pre-existing iD in Editorial Manager. Please see the following video for instructions on linking an ORCID iD to your Editorial Manager account: https://www.youtube.com/watch?v=_xcclfuvtxQ | 0009-0002-3623-3749 |
| 5. Your ethics statement should only appear in the Methods section of your manuscript. If your ethics statement is written in any section besides the Methods, please delete it from any other section. | Done |
| 6. Please review your reference list to ensure that it is complete and correct. If you have cited papers that have been retracted, please include the rationale for doing so in the manuscript text, or remove these references and replace them with relevant current references. Any changes to the reference list should be mentioned in the rebuttal letter that accompanies your revised manuscript. If you need to cite a retracted article, indicate the article’s retracted status in the References list and also include a citation and full reference for the retraction notice. | Same as previous version |
| **Reviewer #1: Explain novelty of this work.’**  How this work is different from other glipizide sustained release microparticles.  Kindly add more value to paper by adding oral safety parameters with these polymers.  Drug release studies figure needs formatting. Mention your pH used according to time intervals in figure | Unlike previous studies on glipizide sustained-release formulations, which largely focus on first-order release kinetics and limited control over extended release, our work employs a unique combination of Eudragit S100 and Methocel K100M polymers. This facilitates a prolonged drug release for up to 12 hours following a non-Fickian diffusion mechanism. Furthermore, in-vivo studies demonstrated sustained hypoglycemic effects over 8 hours, presenting a significant improvement in the control of drug release.  Done, added in Conclusion section,  Again recreated and mentioned the pH |
